# A Practical Treatise on Massage—Its History—Mode of Application and Effects

**Published:** 1885-01

**Authors:** 


A PRACTICAL TREATISE ON MASSAGE, ITS HISTORY, MODE OF APPLICATION AND EFFECTS.—Indications and counter indications, with results in over fourteen hundred cases. By Douglas Graham, M. D., Fellow of the Massachusetts Medical Society. New York: William Wood & Co., 1884.

After the usual prolixity in recounting the past history of the subject to be treated, the author enters in earnest upon the mode of applying massage in the third chapter, and gives us the minute details of this treatment upon the different portions of the body and the extremities, even to the qualifications and the requisites for a good manipulator.

Afterwards, the physiological effects of massage are taken up, illustrating its influence over the various functions with increased peristaltic action of the bowels. In the fifth chapter, upon neurasthenia and anemia of women, reference is made to the views of numerous practitioners, in different departments and specialties, to support the indications for massage; and the inappositeness of many of these quotations will be comprehended when he takes an extract from Dr. T. Gaillard Thomas, favoring "a sea voyage, a visit to a watering place, or a few months passed in the country," and follows it by the remark, "When these fail, try massage." This manner of pressing into service the names of prominent men of the profession cannot supply the lack of reliable data in illustration of rubbing down for the various disorders of the animal economy; and its application is urged, even to the head, face, eyes, ears and throat, as effective.

While the results claimed encourage the resort to massage in some classes of disorders, it should not be expected to cover all diseases; and the future prospects of massage depend upon its proper limitation.

